# Toward Sustainable Printed Packaging: Surface Properties and Ink Adhesion Behavior of PLA/PCL/Nanosilica Biopolymer Blends

**DOI:** 10.3390/polym18030422

**Published:** 2026-02-06

**Authors:** Sanja Mahović Poljaček, Tamara Tomašegović, Dino Priselac

**Affiliations:** University of Zagreb Faculty of Graphic Arts, Getaldićeva 2, 10000 Zagreb, Croatia; dino.priselac@grf.unizg.hr

**Keywords:** PLA/PCL blend, nanosilica, morphology, wetting behavior, ink adhesion

## Abstract

In this study, polylactic acid (PLA) was blended with poly(ε-caprolactone) (PCL) and reinforced with nanosilica (SiO_2_) to tailor surface characteristics and improve adhesion in biopolymer-based printed packaging applications. The surface microstructure and topography were analyzed using FTIR-ATR, SEM, and surface profilometry. Surface wettability and surface free energy (SFE), along with the adhesion properties of printed ink layers on polymer blends, were assessed, and the optical properties of the substrates and prints were evaluated. SEM revealed that PLA/PCL blends exhibited phase-separated morphologies with PCL droplet domains, whereas incorporation of 3 wt% SiO_2_ resulted in finer dispersion and reduced surface irregularities. Surface roughness (Ra) increased from 1.92 µm for PLA/SiO_2_ 100/3 to 4.45 µm for PLA/PCL/SiO2 50/50/0, while water contact angle decreased from 70.9° for neat PLA to 43.4° for PLA/SiO_2_ 100/3 surface, reflecting enhanced hydrophilicity. SFE components ranged from 26 to 40.7 mJ/m^2^ (dispersive) and 3.2 to 21.5 mJ/m^2^ (polar). Adhesion parameters (interfacial tension ranging from 0.01 to 5.54 mJ/m^2^, work of adhesion from 76.9 to 97.3 mJ/m^2^, and wetting coefficient from 3.04 to 11.1 mJ/m^2^) indicated favorable ink compatibility for most blends, and optical density of the printed layers (1.85–2.35) confirmed potential for good printability. These findings demonstrate that PLA/PCL/SiO_2_ blends allow controlled tuning of surface morphology, wettability, and adhesion, providing a promising approach for biodegradable and print-ready packaging substrates.

## 1. Introduction

The growing demand for environmentally sustainable packaging materials has driven intensive research into biodegradable polymer systems as alternatives to conventional plastics. Environmentally sustainable packaging materials aim to reduce environmental impact throughout their entire life cycle, from raw material extraction to disposal or reuse. As concerns about climate change, resource depletion, and plastic pollution increase, sustainable packaging has become an essential focus for businesses and consumers alike [1].

One key category of sustainable packaging is renewable materials, such as paper, cardboard, bamboo, and plant-based fibers [2,3]. These materials are derived from resources that can be replenished naturally, often with a lower carbon footprint than fossil-fuel-based plastics. When responsibly sourced and certified, they support sustainable forestry and agriculture while remaining widely recyclable or biodegradable [4].

Another important group is recycled and recyclable materials. Packaging made from recycled paper, glass, metals, or plastics reduces the demand for virgin raw materials and lowers energy consumption during production. Designing packaging that is easy to recycle—especially when using single materials, minimal additives, and clear labeling—helps keep materials in circulation and supports a circular economy [5,6,7].

Biodegradable and compostable materials are also gaining attention. These include bioplastics made from cornstarch, sugarcane, or cellulose, which can break down under specific conditions. When managed properly through industrial or home composting systems, these materials can reduce long-term waste accumulation [8]. Common examples include polylactic acid (PLA), produced from fermented cornstarch or sugarcane, and cellulose-based films derived from wood pulp or agricultural residues [9]. These materials can provide similar transparency, rigidity, and processability to traditional plastics, making them suitable for packaging applications such as food containers, cups, and films. Under controlled conditions, typically in industrial composting facilities with regulated temperature, humidity, and microbial activity, many of these bioplastics can biodegrade into water, carbon dioxide, and biomass. However, their environmental benefits depend heavily on proper disposal and appropriate waste management infrastructure, as they may not break down effectively in natural environments or conventional recycling systems [10,11].

Polylactic acid (PLA) is one of the most widely used bioplastics in the packaging industry [9,12,13]. It is an aliphatic polyester derived from renewable resources such as cornstarch or sugarcane and offers good transparency, gloss, and stiffness, making it an attractive alternative to conventional plastics like PET (polyethylene terephthalate) and PS (polystyrene) [14,15]. PLA exhibits relatively high tensile strength (50–70 MPa), Young’s modulus (~3.0 GPa), and good transparency, making it suitable for rigid and semi-rigid packaging, including bottles, films, trays, and disposable containers. However, PLA has some limitations, such as low thermal resistance (glass transition temperature around 60 °C) and brittleness, which can restrict its use in certain packaging applications. To address these limitations, PLA is often blended with another polymer or a nanoscale filler to customize the properties of the resulting blend [16]. For example, PLA is commonly mixed with polymers such as polycaprolactone (PCL) to overcome PLA’s inherent brittleness and low toughness. PCL is a synthetic aliphatic polyester recognized for its excellent flexibility, toughness, and biodegradability under both aerobic and anaerobic conditions [17,18]. It has a low melting temperature (approximately 60 °C) and a glass transition temperature around −60 °C, resulting in high flexibility and elongation at break (>700%). Despite these advantages, its low mechanical rigidity, softness, and relatively high cost have limited its use as a standalone material in the packaging sector.

However, PCL can serve as a valuable blend component to enhance the overall performance of other biodegradable polymers, such as PLA. Numerous studies are available on blending PLA with PCL, mainly focused on investigating its mechanical, thermal, and biodegradable properties. In research on PLA/PCL blends [19], it was found that non-compatibilized PLA/PCL blends containing a small amount of PCL increased the ductility of PLA, whereas higher PCL content was necessary to achieve a significant improvement in impact strength. Morphological analysis showed that the enhanced impact performance was associated with a transitional morphology between sea–island and co-continuous structures in the observed blend. The authors of [20] studied blends of PCL and PLA as materials for the production of relief printing plates used in embossing. Thermal analysis and interfacial interaction studies confirmed the immiscibility of PLA and PCL. Efforts to enhance the impact strength of PLA have focused on increasing its crystallinity and refining the spherulitic morphology of the PLA matrix through the incorporation of PCL. The mechanical properties of PLA/PCL blends have been reported in the literature [21], where PCL addition was shown to improve the impact strength of PLA by promoting higher crystallinity and a finer spherulitic structure. Similar investigations into PLA/PCL blends were conducted in [22], demonstrating that PCL enhances the ductility of PLA, as shown by increased elongation at break. Despite the inherent immiscibility of these polymers, improvements in ductile behavior were achieved without the use of compatibilizers or additives. Morphology and mechanical properties of PLA/PCL blends were studied in [23]. It was found that the proper choice of PLA and PCL components, combined with specific processing conditions (melt-mixing, compression molding, fast cooling), influenced the morphology and toughness of the blends. Properties of PLA/PCL films were studied in [24], where it was found that the tensile strength and elongation at break of PLA/PCL films without additives slightly increased with higher PCL content, confirming overall studies. Furthermore, several research studies on PLA/PCL blends focus on developing materials that can be extruded into 3D printing filaments for use in fused filament fabrication (FFF) or related additive manufacturing methods. These studies, including the one mentioned earlier, mainly consider mechanical properties, thermal behavior, blend compatibility, and the possibility for use in 3D printing [25,26,27,28].

It is worth noting that most of these studies examined the influence of adding fillers or nanoparticles to the PLA/PCL blend. Due to the partial immiscibility of PLA and PCL, a third component has been used in the mixture to enhance interfacial adhesion and achieve optimal mechanical performance in the resulting blends [29]. Compatibilizers such as maleic anhydride (MAH), glycidyl methacrylate (GMA), or block copolymers (e.g., PLA-b-PCL) have been used to significantly improve the morphological homogeneity and overall toughness of the blend [30,31]. Additionally, commonly used nanomaterials as compatibilizers in two- or three-component matrices include graphene oxide, carbon black, carbon nanotubes, nanosilica, fullerenes, and others [32,33].

In this research, PLA was blended with PCL and reinforced with nanosilica (SiO_2_) to create a new, sustainable packaging material with tailored properties. The successful development of novel packaging materials depends on comprehensive characterization of their physicochemical, mechanical, and barrier properties, as well as an evaluation of their interactions with printing inks and printing processes. The interaction between biodegradable packaging materials and printing inks is crucial for ink adhesion, print durability, and potential ink migration, all of which directly affect packaging performance and product safety. Despite its importance and the growing interest in biodegradable materials as sustainable packaging solutions, this aspect remains insufficiently explored, and to our knowledge, only a few studies have addressed it. In [34], the surface, barrier, and colorimetric properties of two plastic films (polypropylene and polyethylene terephthalate) and two biodegradable films (PLA and cellulose) were investigated. Results showed that the optical density of inks printed on biodegradable films was comparable to that on traditional plastic films. It was confirmed that the type of film determined the print quality. The study concluded that films based on PLA and cellulose can successfully serve as alternatives to traditional polymers, but further research was suggested to examine other material properties in detail.

Other studies have examined the flexographic printing performance of PLA films [35]. These studies found that PLA films were comparable in printability and runnability to standard petroleum-based flexible packaging films. Research on the printability of PLA films compared the print qualities to common packaging films such as low-density polyethylene (LDPE), corona-treated LDPE, and polyethylene terephthalate (PET) was published [36]. The surface properties of the films, ink adhesion to the substrate, and optical density of printed samples were evaluated. It was concluded that PLA can be successfully printed with flexographic solvent-based inks and can achieve quality similar to common packaging films. Results on the influence of surface modification on the print quality of biodegradable PLA films were published in [37]. Unmodified and modified films were printed in flexography, and the quality of the prints was analyzed. Surface, mechanical, and colorimetric properties were measured. The results showed a strong effect of surface modification on the properties of the films, suggesting that the use of plasma surface modification in PLA films can improve printability.

The results of the abovementioned studies showed the possibilities of printing on pure PLA, despite its properties, which in some cases do not meet the performance requirements for practical applications. According to the existing literature, which mainly focuses on neat PLA, there is an identified research gap regarding the printing possibilities of neat PLA compared to its blends, which can address the shortcomings of neat PLA. To expand the potential packaging applications of neat PLA, this study analyzed the printing possibilities of biodegradable polymer blends based on PLA, with the addition of PCL and silica (SiO_2_) nanoparticles. Specifically, the aim of this research was to investigate the interactions between newly developed PLA/PCL blends and printing inks, with particular emphasis on surface and blending properties, the influence of nanofiller addition, ink adhesion, and print performance. PLA was used in this research as the matrix for producing biodegradable blends. To reduce its brittleness, which limits its use in some applications, and to modify the surface properties of the blend, PCL was added in varying amounts. Additionally, silica was added to examine the effect of the filler on the miscibility and properties of the resulting new materials. It was assumed that the properties of the produced blends could be tailored by adjusting the PLA/PCL ratio or by incorporating a compatibilizer to enhance interfacial adhesion between the two polymers and improve the printability of the materials. The morphological and surface properties of the blends, the interaction of the polymer materials with printing ink, and the optical properties of the prints were investigated. Therefore, the present work systematically analyzes the morphology, surface structure, roughness, wettability, and interfacial adhesion of PLA/PCL/SiO_2_ blends with flexographic inks, providing new insights into the design of biodegradable substrates for printing applications.

## 2. Materials and Methods

### 2.1. Materials

PLA Ingeo™ 3251D (NatureWorks LLC, Plymouth, MN, USA) was used as the main polymer matrix. PLA was selected for this research because it is widely used in packaging applications and is suitable for overprinting with flexographic printing technology. PLA has a glass transition temperature of 50–60 °C, a melting temperature of about 170 °C, a tensile strength of 60 MPa, and an elongation at break of 3.5% [12]. Poly(ε-caprolactone) (PCL Capa™ 6800, Perstorp, Warrington, UK) was added to the PLA matrix to improve the flexibility of the resulting blend. PCL has a glass transition temperature of −60 °C, a melting temperature of 60 °C, a tensile strength of 20 MPa, and a tensile elongation of approximately 800% [17]. PLA/PCL blends were prepared with PCL contents up to 50 wt%, resulting in compositions of PLA/PCL 100/0, 90/10, 80/20, 70/30, 60/40, and 50/50.

Fumed silica (Aerosil^®^ 200, Evonik, Hanau, Germany) with an average particle size of 12 nm was used as received, without any pre-treatment. To study the effect of nanosilica, additional mixtures with different PLA/PCL ratios were prepared containing 1 wt% and 3 wt% silica. Silica nanoparticles were introduced into the mixtures to influence interfacial interaction, dispersion, and overall performance of the PLA/PCL blends.

**Figure 1:**
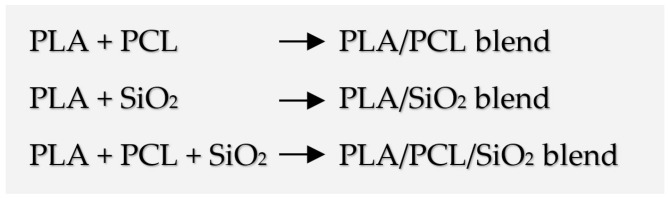
Schematic flow diagram of blending PLA with PCL and SiO_2_.

The prepared samples included neat PLA, blends with varying proportions of PLA and PCL (up to 50 wt%), and samples with the addition of 1 wt% and 3 wt% silica. The samples were labeled according to their PLA, PCL, and SiO_2_ content: PLA 100 (neat PLA), PLA/PCL 90/10 (90 wt% PLA and 10 wt% PCL), PLA/PCL 80/20 (80 wt% PLA and 20 wt% PCL), and so on. Samples containing nanosilica were labeled as PLA/SiO_2_ 100/1 and PLA/SiO_2_ 100/3 (neat PLA with 1 wt% and 3 wt% silica), PLA/PCL/SiO_2_ 90/10/1 (90 wt% PLA, 10 wt% PCL, and 1 wt% SiO_2_), PLA/PCL/SiO_2_ 90/10/3 (90 wt% PLA, 10 wt% PCL, and 3 wt% SiO_2_), and so on. All blends were compounded using a Brabender^®^ (Duisburg, Germany) internal mixer at 190 °C for 5 min. The homogeneous melts were then compression molded into thin plates (100 × 100 × 1 mm) using a hydraulic press at 190 °C and 16 MPa for 7 min, including preheating. Figure 1 shows a schematic flow diagram of mixing PLA with PCL and SiO_2_ components.

A UV-curable black ink for flexographic printing (Sun Chemical Group, Weesp, Netherlands) was applied to each polymer blend. The ink layer was deposited using an Elcometer 3580 casting knife film applicator (Elcometer Limited, Manchester, UK). After ink coating, the samples were cured in a Technigraf Aktiprint L 10-1 UV dryer (Technigraf GmbH, Hessen, Germany) at a speed of 4 m/s, with five passes under UV radiation. The UV source in the dryer has an emission range of 210–380 nm (2870 ± 5 mW/cm^2^) and 320–420 nm (1150 ± 20 mJ/cm^2^). After a stabilization period of 24 h, all measurements and analyses were performed.

### 2.2. Methods

Fourier transform infrared spectroscopy with attenuated total reflectance (FTIR-ATR) was used to investigate the chemical composition and molecular structure of the samples, as well as to assess structural variations associated with different PLA/PCL ratios and varying contents of SiO_2_ nanoparticles. The measurements were carried out using an IRAffinity-1 FTIR spectrophotometer (Shimadzu, Kyoto, Japan) equipped with a ZnSe crystal (refractive index 2.4). Spectra were collected with 15 scans at a resolution of 4 cm^−1^ over the wavenumber range of 3600–600 cm^−1^.

To observe the surface morphology of the produced materials, a scanning electron microscope (SEM) (JSM-6060LV, Jeol, Tokyo, Japan) was used. Before imaging, the samples were coated with a thin gold layer using a high-vacuum evaporation process.

The surface structure of the produced materials was measured with a MarSurf PS 10 gauge (Mahr GmbH, Göttingen, Germany) using the stylus method. Analysis of the basic roughness parameters provides insight into how the preparation conditions of polymer blends affect the surface of polymeric materials. This analysis also provides detailed information about the surface structure resulting from the addition of PCL and silica nanoparticles to the PLA matrix. Three roughness parameters were measured: R_a_, the arithmetic mean deviation of the surface profile; R_z_, the vertical distance between the highest peak and the deepest valley within the measuring length; and the maximum roughness depth (R_max_), the largest single roughness depth within the evaluation length, according to ISO 21920-2:2021 [38]. The stylus size was 2 µm, and the measuring force was 0.00075 N. Each sample was measured ten times, and the average value was calculated.

To observe the wettability of polymeric blends, the contact angle of water was measured over time on produced PLA/PCL/SiO_2_ blends. Wetting of a solid surface by water refers to water’s ability to spread or bead on the surface, determined by the balance between adhesive forces (attraction between water and the solid) and cohesive forces (attraction between water molecules). Wettability with water is an important parameter for characterizing newly produced printing substrates because it reflects the surface free energy and polarity of the material, which directly influence ink spreading, adhesion, and drying behavior. Wettability was measured using a DataPhysics OCA 30 goniometer (DataPhysics Instruments GmbH, Filderstadt, Germany).

To further characterize the surface properties of the produced materials, the surface free energy (SFE) was calculated using the Owens–Wendt method. This widely used method determines the SFE of a solid by measuring contact angles with different liquids and dividing the SFE into its dispersive (nonpolar) and polar (hydrogen bonding, acid–base) components, based on geometric mean assumptions about intermolecular forces. By plotting data from liquids with known surface tension using Young’s equation, the solid’s polar and dispersive SFE components can be determined from the slope and intercept of a straight line, revealing surface chemistry and properties such as liquid repellency or adhesion [39,40]. Contact angle measurements were repeated five times for each liquid and coating surface. The average contact angle values and standard deviations are reported. Environmental conditions were maintained at 23 ± 1 °C. In this study, water, diiodomethane, and glycerol were used as reference liquids. Their properties are shown in Table 1.

Contact angle measurements of probe liquids on the polymer surfaces were performed using a DataPhysics OCA 30 goniometer (DataPhysics Instruments GmbH, Filderstadt, Germany). Surface free energy was calculated using SCA20 software for OCA and PCA (DataPhysics Instruments GmbH). Additionally, adhesion between the printing ink layer and polymer surfaces was calculated. Three parameters indicating possible adhesion were determined: thermodynamic work of adhesion (W_12_), wetting coefficient (S_12_), and interfacial tension (γ_12_) [39]. For optimal adhesion, the thermodynamic work of adhesion should be maximal, the wetting coefficient should be close to zero, and the interfacial tension should be positive or zero [40].

To determine the optical density of the produced polymeric blends and the applied ink layer, a film transmission and color reflection densitometer was used (Techkon DENS, Techkon GmbH, Königstein, Germany). The optical density of the polymeric blends was measured to assess the optical properties of the produced materials. The optical density of the ink layer was measured to evaluate the print quality achieved with the flexographic ink on the produced polymer substrates. Calibration was performed on paper white for measuring the optical density of the PLA/PCL/SiO_2_ blends and on polymer substrates for measuring the optical density of the ink layer. The thickness of the printed ink layer was measured using a DUALSCOPE^®^ FMP100 device (Helmut Fischer GmbH, Sindelfingen, Germany), which operates using the magnetic induction test method. The average of five measurements taken for each sample is reported.

## 3. Results

### 3.1. FTIR-ATR Spectra of PLA/PCL/SiO_2_ Blends

**Figure 2:**
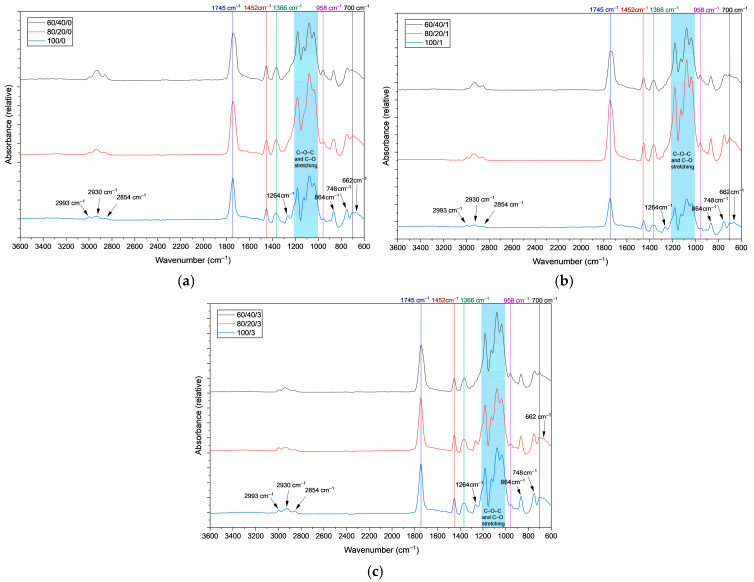
FTIR-ATR spectra of PLA/PCL/SiO_2_ blends: (**a**) without SiO_2_; (**b**) with 1 wt% SiO_2_; (**c**) with 3 wt% SiO_2_.

FTIR-ATR spectroscopy was used to examine neat PLA, PLA/PCL blends without SiO_2_, and PLA/PCL/SiO_2_ blends to assess the influence of the PLA/PCL ratio and nanosilica addition on potential changes in chemical structure. Selected FTIR-ATR spectra are shown in Figure 2a–c. As no new chemical bonds characteristic of specific spectra were detected following the modification of the blend composition, the presented spectra illustrate the trends and changes occurring in the films with variations in component ratio.

The spectrum of neat PLA (100/0) (Figure 2a) shows absorption bands characteristic of poly(lactic acid). The strong band at 1743 cm^−1^ is attributed to ester carbonyl (C=O) stretching vibrations, typical for PLA [41,42]. Bands at 1452 cm^−1^ and 1366 cm^−1^ correspond to asymmetric and symmetric bending vibrations of CH_3_ groups in the PLA backbone [41,42]. The absorption at ~1264 cm^−1^ is assigned to asymmetric C–O–C stretching of the ester bond, while the bands at around 1181 cm^−1^, 1126 cm^−1^, and 1037 cm^−1^ can be attributed to C–O–C and C–O stretching vibrations of the ester groups in PLA [43,44,45]. The lower-wavenumber bands at 864 cm^−1^ and 748 cm^−1^ can be associated with C–C skeletal vibrations and CH deformation modes of PLA [46].

Incorporation of PCL into PLA (80/20/0 and 60/40/0) (Figure 2a) leads to subtle changes in the spectra, reflecting the increasing contribution of PCL. The band at ~958 cm^−1^, which is increasing in intensity with higher PCL content, can be attributed to C–C skeletal vibrations associated with crystalline PCL [47].

The band at 1264 cm^−1^, prominent in neat PLA, decreases with increasing PCL content, reflecting the decreased intensity of PLA-specific ester vibrations. The 1366 cm^−1^ band, present in all compositions, becomes narrower with increasing PCL content, which can be attributed to the growing influence of PCL CH_2_ bending modes overlapping this region (Figure 2a–c). In the high-wavenumber region, the CH stretching bands at 2854 cm^−1^, 2930 cm^−1^, and 2993 cm^−1^ become more pronounced with increasing PCL content due to the higher methylene group concentration in PCL compared to PLA [42]. The continuous presence of the carbonyl band at ~1743 cm^−1^, without a significant shift except subtle widening at higher PCL concentrations, and the absence of new absorption bands indicate that no chemical reactions occur between PLA and PCL, confirming that the blends are formed primarily through physical mixing.

The influence of nano-SiO_2_ was examined for samples containing 1 wt% and 3 wt% silica (Figure 2b,c) compared to the silica-free samples (Figure 2a). Overall, the FTIR-ATR spectra of silica-containing samples are similar to those of the corresponding silica-free blends, indicating that the addition of SiO_2_ does not alter the primary chemical structure of the polymer matrix. A weak band around ~662 cm^−1^, observed in some spectra, may be attributed to methyl (–CH_3_) group rocking or wagging modes and skeletal vibrations of the polymer backbone, since the weaker absorptions in the ~700–650 cm^−1^ region are commonly assigned to these types of vibrations [46]. Bands near ~700 cm^−1^, 748 cm^−1^, and 864 cm^−1^ are observed in all silica-containing samples and can also be associated mainly with polymer backbone vibrations [46]. The ester-related bands at 1126 cm^−1^ and 1181 cm^−1^ remain present in all samples, regardless of the specific polymer content or nano-SiO_2_ concentration. The band at 1264 cm^−1^ shows higher absorbance in the neat PLA samples compared to PCL-containing compositions, reflecting the higher PLA content. The 1366 cm^−1^ band is broader in the neat PLA samples than in the blends.

In summary, FTIR-ATR analysis confirms that the addition of PCL and nano-SiO_2_ does not result in a chemical modification of the PLA matrix. The observed spectral changes are primarily related to compositional effects and physical interactions. Increasing PCL content leads to the increased appearance and growth of characteristic PCL bands, accompanied by a reduction in PLA-dominant vibrations, while nano-SiO_2_ mainly influences band widths and relative intensities, indicating that no new chemical bonds were formed.

### 3.2. Morphology, Roughness, and Surface Profiles of PLA/PCL/SiO_2_ Blends

**Figure 3:**
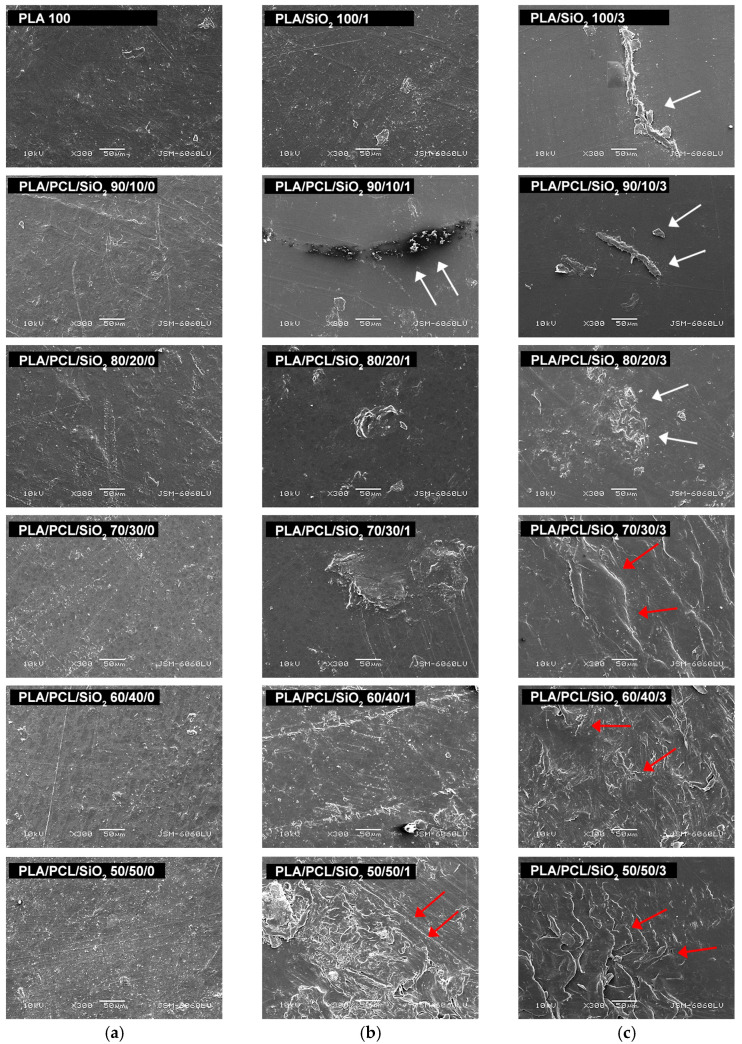
SEM micrographs of PLA/PCL blends: (**a**) without SiO_2_; (**b**) with 1 wt% SiO_2_; (**c**) with 3 wt% SiO_2_ (mag. 300×) (white arrows indicate accumulations of material on the polymer base surface; red arrows indicate slight delamination of the materials).

The morphology of PLA/PCL/SiO_2_ blends was examined to determine the surface structures of biodegradable polymeric materials. SEM images of all blends are shown in Figure 3.

As shown in the image, the surfaces of PLA/PCL samples without nanoparticles appeared relatively uniform. Adding PCL to the PLA matrix did not cause visible changes in surface morphology. Adding nanoparticles at 1 wt% and 3 wt% also did not alter the surface structure. However, differences in surface contrast indicated changes in surface properties, particularly in images with lower PCL content, where separate accumulations of material are visible on the polymer base surface (especially in PLA/PCL/SiO_2_ samples: 100/0/3, 90/10/1, and 90/10/3). In samples containing 30 wt% PCL or more, uneven surface structures were observed, which may have resulted from minimal delamination of the material in these mixtures, as previously reported [48].

**Figure 4:**
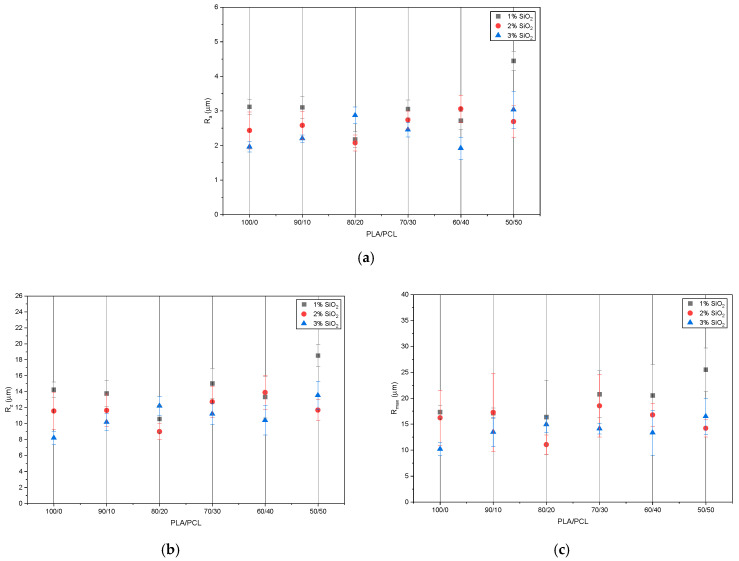
Roughness parameters: (**a**) R_a_; (**b**) R_z_; (**c**) R_max_.

To examine the surface structure of the samples in more detail, roughness parameters R_a_, R_z_, and R_max_ were measured, and the results are presented in Figure 4.

Figure 4 shows the measured values of the three basic roughness parameters. The average values after ten measurements are presented, with five taken in the *X* direction and five in the *Y* direction of the sample. The results indicate that the roughness values for all three parameters increased with the addition of PCL to the mixture. When the results are considered individually, the following conclusions can be drawn: The R_a_ parameter (Figure 4a), which represents the average absolute value of the profile deviation from the center line, ranged from 1.92 µm (the lowest value measured on the PLA/PCL/SiO_2_ 80/20/3 sample) to 4.45 µm (the highest value measured on the PLA/PCL/SiO_2_ 50/50/0 sample). In general, in all samples, the addition of PCL resulted in an increase in the R_a_ parameter of 0.5 to 1 µm. Notably, the addition of silica to the PLA/PCL blend also changed the surface structure of the material. According to the measurements, higher roughness values were recorded on samples without silica (0 wt% SiO_2_) than on those with added silica. Specifically, the lowest roughness values were measured on samples containing 3 wt% SiO_2_. The exception was the PLA/PCL 80/20 sample, where the roughness values remained nearly constant regardless of the addition of nanoparticles.

The results of the Rz roughness parameter measurements are shown in Figure 4b. This parameter represents the mean height of the roughness, calculated as the average of the five highest peak heights and the five deepest valley depths within the measurement length. The values for the samples ranged from 8.19 mm to 18.53 µm. As observed with the Ra parameter, adding PCL as the second component in the mixture increased the roughness height. The smallest roughness was recorded on the PLA sample with 3 wt% silica added (PLA/SiO_2_ 100/3), while the largest was found on the PLA/PCL 50/50 sample. It is also noted, as with the Ra parameter results, that the addition of nanoparticles partially reduced the height of the surface irregularities. The addition of 3 wt% silica generally decreased the mean asperity heights, except for the PLA/PCL 80/20 sample, as also seen in the Ra parameter measurements.

The results for the Rmax roughness parameter are shown in Figure 4c. The Rmax parameter describes the maximum height of surface irregularities, defined as the largest distance between the highest peak and the deepest valley within the measured length. According to the measurements, the maximum heights of irregularities on the samples ranged from 10.19 µm to 25.51 µm. These results were consistent with those for the Ra and Rz parameters. The addition of PCL to the mixture caused a slight increase in the height of surface irregularities in the polymer blends. Higher irregularity heights were observed in samples without nanoparticles compared to those with nanoparticles. Based on these results, it can be concluded that the addition of nanoparticles positively affects the surface of polymer blends by reducing roughness.

**Figure 5:**
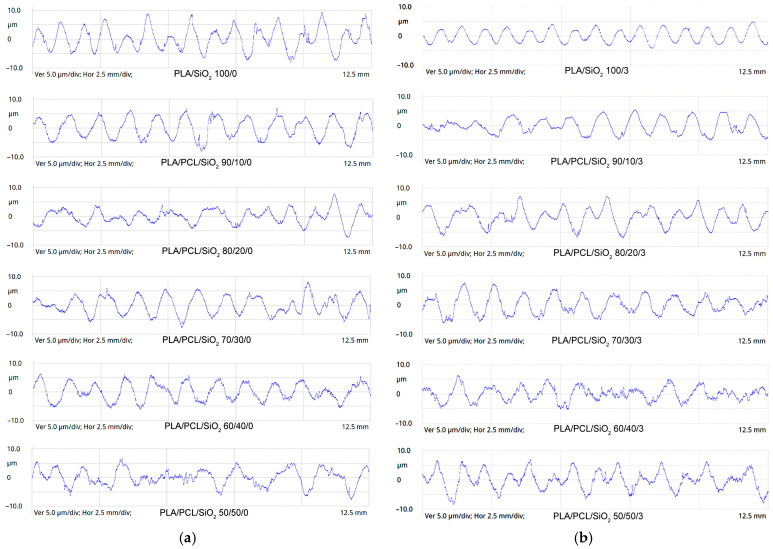
Surface profiles of PLA/PCL blends: (**a**) without SiO_2_; (**b**) with 3 wt% SiO_2_.

To further examine the surface structures of the PLA/PCL/SiO_2_ blends, surface profiles were measured, and the surface profiles of selected blends are shown in Figure 5. The appearance of these surface profiles corresponds with the roughness parameter results shown in Figure 4. The irregularities in the surface structure of the blends increased with the addition of the PCL component to the PLA matrix. Adding PCL resulted in greater irregularity in the surface profile, particularly in the height of peaks and valleys. The images also show the surface profiles of samples containing 3 wt% nanoparticles, which display a noticeable reduction in surface irregularities. The surface profiles of all samples exhibit fewer irregularities, especially at the tops of the peaks and the deepest parts of the valleys. The reduction in surface roughness was detected and confirmed by measurements of the R_a_, R_z_, and R_max_ roughness parameters.

The increase in surface roughness observed with the incorporation of PCL into PLA can be attributed to the intrinsic immiscibility and phase separation of the PLA/PCL blend. Numerous studies have shown that PLA and PCL form heterogeneous blends in the melt, resulting in PCL-rich domains dispersed within the PLA matrix, which manifest as surface irregularities after cooling and solidification [48,49,50]. Additionally, PLA and PCL have different crystallization kinetics and melting temperatures, with PCL crystallizing more rapidly and at lower temperatures, promoting localized crystalline structures and surface topography variations during the cooling process [51]. Differences in melt viscosity and elastic recovery between the two polymers during hot pressing further contribute to flow instabilities and surface deformation. Moreover, PCL has lower surface free energy and higher chain mobility than PLA, favoring surface segregation of PCL, which has been reported to increase surface roughness and heterogeneity in PLA/PCL blends [28,52].

Incorporating silica nanoparticles into the blends resulted in reduced surface roughness, indicating a more stable and homogeneous surface morphology. This effect can be attributed to the role of silica nanoparticles as physical compatibilizers, which suppress phase coalescence by localizing at phase interfaces, thereby reducing interfacial tension and domain size [28]. Additionally, the presence of silica nanoparticles modifies melt rheology and restricts polymer chain mobility, leading to more uniform flow during mixing and melting, and limiting surface instabilities and differential shrinkage upon cooling. Furthermore, nanoparticle-induced confinement and nucleation effects can produce finer and more evenly distributed crystalline structures, which collectively contribute to the smoother surface profile of silica-filled PLA/PCL blends compared to blends without silica [29].

### 3.3. Cross-Sections of PLA/PCL/SiO_2_ Blends

**Figure 6:**
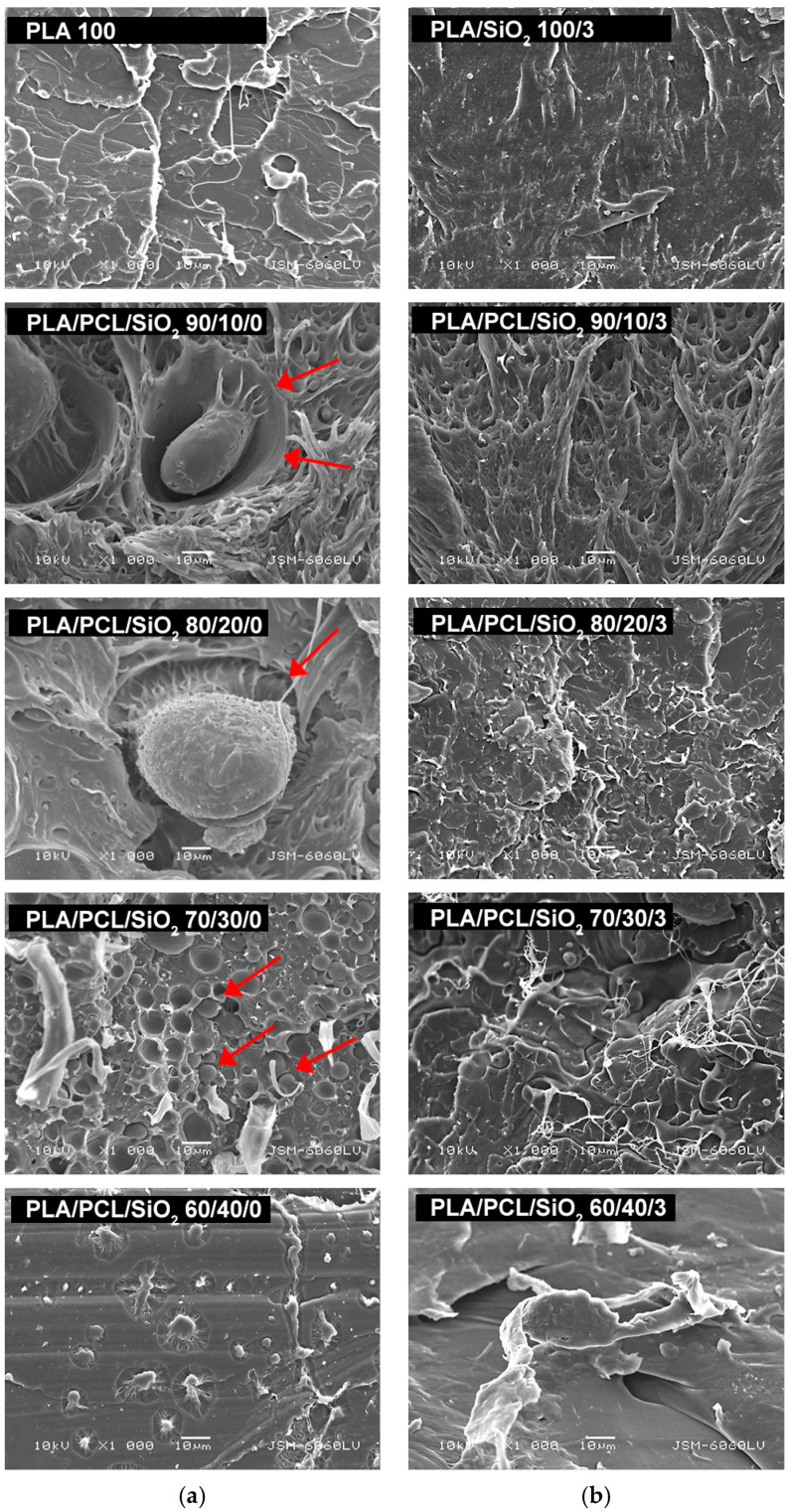
SEM micrographs of cross-sections of PLA/PCL blends: (**a**) without SiO_2_; (**b**) with 3 wt% SiO_2_ (mag. 1000×) (red arrows indicate spherical PCL domains incorporated into the PLA matrix).

Additional analysis of the distribution of components and surface structures in the PLA/PCL/SiO_2_ blends was conducted using cross-sections of selected samples. Scanning electron microscopy images are shown in Figure 6. The cross-sections of PLA/PCL samples are presented both without silica nanoparticles and with 3 wt% added silica.

Cross-sections of blends without added SiO_2_ nanoparticles (Figure 6a) showed a coarse morphology (also referred to as droplet-in-matrix or sea–island structure), in which PCL spherical particles are surrounded by the continuous PLA phase (spherical PCL domains are marked with red arrows and correspond to the typical sea–island morphology). The particles immersed in the PLA matrix fell out of their “beds” upon fracture, indicating poor interfacial adhesion.

In our previous research, where the same materials were used to produce biodegradable polymer printing plates using the same methodology, it was found that PLA/PCL blends exhibit two distinct phases in the surface fracture, with spherical domains of dispersed PCL in PLA [42,48]. The same observation is evident in Figure 6, especially in samples PLA/PCL 90/10, 80/20, and 70/30. The presence of spherical PCL domains dispersed in the PLA matrix arises from the thermodynamic immiscibility between PLA and PCL [53,54,55]. Due to unfavorable intermolecular interactions, phase separation occurs during melt blending, with PLA forming the continuous phase and PCL dispersing as droplets. The spherical morphology of the PCL phase minimizes interfacial free energy and is characteristic of immiscible polymer blends with a minor dispersed component below the phase inversion threshold.

When observing the cross-sections of PLA/PCL blends containing 3 wt% SiO_2_ nanoparticles (Figure 6b), the morphology appeared more irregular and relatively uneven. However, the isolated spherical PCL droplets seen in samples without SiO_2_ were no longer visible. This behavior can be attributed to nanoparticle-induced compatibilization, with SiO_2_ nanoparticles preferentially localizing in the PCL phase, as reported in [42]. As a result, the PCL phase is likely dispersed at a much finer length scale, below the resolution limit of SEM, leading to the detected change in morphology in the cross-sectional images.

Specifically, introducing silica nanoparticles shifts the morphology from a micron-scale phase-separated structure to a nanoscale dispersed morphology, reducing the amplitude of surface height fluctuations. This leads to finer dispersion of the PCL phase and suppresses micron-scale phase protrusions at the surface of the blends. The results are consistent with the surface images and roughness measurements (Figure 3, Figure 4 and Figure 5), which showed that adding PCL increased surface irregularities and roughness. In contrast, incorporating silica into all PLA/PCL blends partially reduces surface irregularities and decreases surface roughness.

### 3.4. Wettability, Surface Free Energy, and Adhesion Properties of PLA/PCL/SiO_2_ Blends

#### 3.4.1. Water Contact Angle

**Figure 7:**
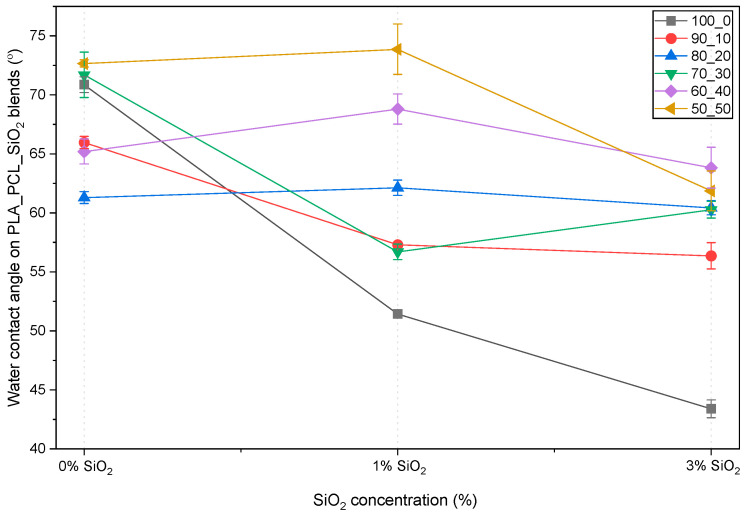
Water contact angle on PLA/PCL/SiO_2_ blends.

The surface wettability of the PLA/PCL/SiO_2_ blends was evaluated by static water contact angle measurements, taken 45 s after the water droplet touched the polymer surface and stabilized. The sessile drop method was applied with a droplet volume of 1 μL. The measured contact angles are presented in Figure 7 (Table S7).

The neat PLA sample (100/0) exhibited an average water contact angle of 70.9°, consistent with the moderately hydrophobic nature of PLA [42]. The incorporation of SiO_2_ led to a pronounced decrease in contact angle, reaching 51.5° for the sample with 1 wt% SiO_2_ (sample 100/0/1) and 43.4° for the sample with 3 wt% SiO_2_ (sample 100/0/3). This significant reduction indicates a noticeable increase in surface hydrophilicity induced by the silica nanoparticles [42]. This behavior can be attributed to the presence of silanol (-Si-OH) groups on the surface of SiO_2_, which would enhance polar interactions with water. In addition, the migration of silica particles towards the polymer–air interface may further increase the hydrophilic nature of the surfaces with silica nanoparticles [56,57,58]. These results demonstrate that SiO_2_ is highly effective in modifying the surface wettability of PLA, even at low concentrations.

The water contact angles of PLA/PCL blends without SiO_2_ did not show a monotonic dependence on PCL content. While the introduction of 10–20 wt% PCL resulted in a decrease in water contact angle (from 70.1° for 100/0 to 60.8° for 80/20/0), higher PCL contents led to an increase in contact angle, with values of 73.9° and 72.4° observed for 70/30/0 and 50/50/0, respectively.

This non-linear trend suggests that surface wettability is influenced not only by the polarity of the PLA and PCL but also by blend morphology and surface composition. Occurrences such as phase separation and selective surface enrichment of one polymer phase are likely responsible for these variations. In particular, the higher contact angles at intermediate and high PCL contents may indicate preferential exposure of PCL-rich domains at the surface, as well as changes in crystallinity [42,59].

The addition of SiO_2_ reduced the contact angle across almost all PLA/PCL/SiO_2_ blends compared to the corresponding blends without SiO_2_. However, the presence and significance of this reduction strongly depended on the PLA_PCL ratio.

At low PCL concentrations (≤20 wt%), the presence of SiO_2_ resulted in a decrease in water contact angle, confirming the dominant influence of silica-induced hydrophilicity. In contrast, as the PCL content increased to 40 wt%, the effectiveness of SiO_2_ in lowering the contact angle was not expressed. The blend with 50 wt% PCL presented an effective silica-induced contact angle reduction.

This behavior suggests a competition between the hydrophilizing effect of SiO_2_ and the tendency of PCL to dominate the surface composition in the blends with higher PCL content. Possible explanations include partial shielding of silica particles by the PCL phase due to the encapsulation, reduced surface exposure of silanol groups, or morphological features arising from phase separation [56]. Therefore, surface wettability in these systems could be controlled by the influence of filler chemistry, polymer–filler interactions, and blend morphology.

Overall, the lowest water contact angle (42.6°) was achieved for PLA containing 3 wt% SiO_2_, highlighting the strong potential of SiO_2_ for surface hydrophilization of PLA-based materials. While PCL incorporation alone produced mixed effects on surface wettability, it clearly moderated the impact of SiO_2_ at higher concentrations. These findings emphasize that the surface properties of PLA/PCL/SiO_2_ systems cannot be predicted solely based on blend composition but must also consider surface segregation phenomena and filler distribution [42]. The tunable wettability achieved through controlled variation of the PLA/PCL ratio and SiO_2_ content may be advantageous for applications where surface interactions are critical, such as packaging, adhesion-sensitive systems, and biomedical devices [42,56].

#### 3.4.2. Surface Free Energy of PLA/PCL and PLA/PCL/SiO_2_ Blends

**Figure 8:**
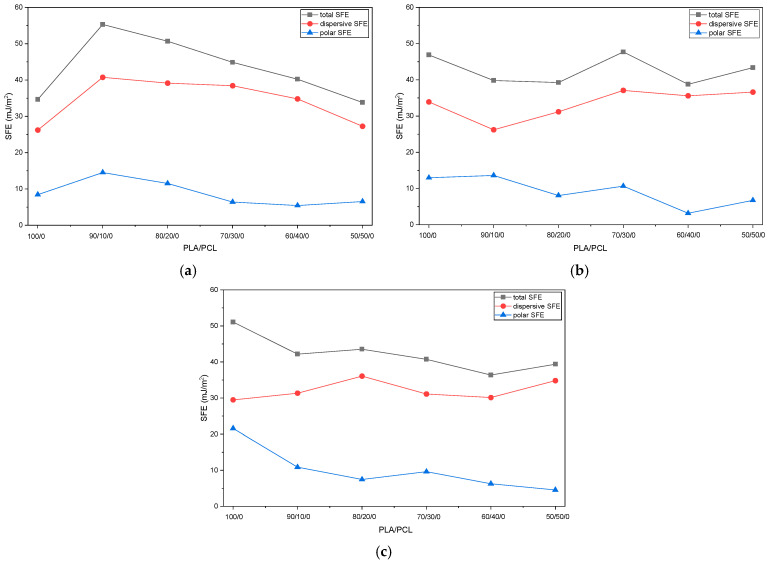
Surface free energy components of PLA/PCL/SiO_2_ blends: (**a**) 0 wt% SiO_2_; (**b**) 1 wt% SiO_2_; (**c**) 3 wt% SiO_2_.

Total, dispersive, and polar surface free energy (SFE) components of PLA/PCL blends with different SiO_2_ content are presented in Figure 8 (Table S8).

Like surface wettability, the SFE of PLA/PCL/SiO_2_ blends is determined by a complex interplay between polymer composition, filler portion, and surface segregation. The total SFE and its dispersive and polar components show non-linear variations with both the PLA/PCL ratio and SiO_2_ content (0, 1, and 3 wt%), reflecting changes in surface chemistry and morphology.

Since SiO_2_ used in this research is a hydrophilic fumed silica with a high specific surface area, its incorporation in PLA leads to a marked initial increase in the polar component of SFE (γᵖ). As already mentioned, this increase can be attributed to the partial exposure of silanol groups at the material surface, as well as to hydrogen bonding interactions between the silica surface and the ester groups of PLA [57].

This effect is most pronounced in blend compositions rich with PLA. For example, in neat PLA systems, increasing SiO_2_ content from 0 to 3 wt% results in a substantial rise in γᵖ, indicating a strong enhancement of surface polarity (Figure 8a–c). In contrast, at 1% wt (Figure 6b), the polar SFE component of blends is often lower than expected, suggesting that SiO_2_ particles are more effectively embedded within the polymer matrix and partially shielded by polymer chains, thereby limiting their direct contribution to SFE.

Despite the high SFE of SiO_2_, the total SFE does not increase monotonically with SiO_2_ concentration across all blend compositions. At low SiO_2_ content (1 wt%), the dominant mechanism appears to be polymer surface segregation rather than SiO_2_ exposure at the material surface. At higher concentration (3 wt%, Figure 8c), increased particle–particle interactions and partial agglomeration of SiO_2_ could enhance the silica protrusion at the surface, leading to a higher polar SFE component but, in some cases, a reduction in the dispersive component [42].

These competing effects explain why some blends exhibit lower total SFE at 1 wt% SiO_2_ compared to both 0% and 3 wt% systems. Furthermore, in all blends, γᵈ constitutes the dominant component of the total SFE, which is typical for polymer-based materials.

An increase in SiO_2_ content generally led to a slight reduction in γᵈ, which can be attributed to the partial replacement of polymer–air interfaces with polymer–silica or silica–air interfaces, as well as increased surface heterogeneity. Blend compositions rich in PCL show a stronger dominance of the dispersive component, probably also due to the lower polarity and higher chain flexibility of PCL [60,61].

Blends with intermediate PLA/PCL ratios (e.g., 70/30 and 60/40) show less systematic trends in SFE. These systems are likely characterized by more complex phase morphologies, including co-continuous or finely dispersed structures [62]. SiO_2_ particles could therefore also localize within one polymer phase, specifically PCL [42], and small changes in filler concentration could significantly affect local chemical composition and, by extension, surface properties. As a result, irregular variations in total and polar SFE are observed.

Overall, the results demonstrate that controlled adjustment of the PLA/PCL ratio and SiO_2_ content could provide an effective strategy to tailor the surface free energy and thereby the wettability, printability, and adhesion properties of biodegradable PLA/PCL/SiO_2_ polymer blends.

To compare the surface properties of the prepared blends with those of commonly used polymer materials in packaging printing, the most common commercial printing substrates were selected, and their surface free energy components were analyzed.

**Figure 9:**
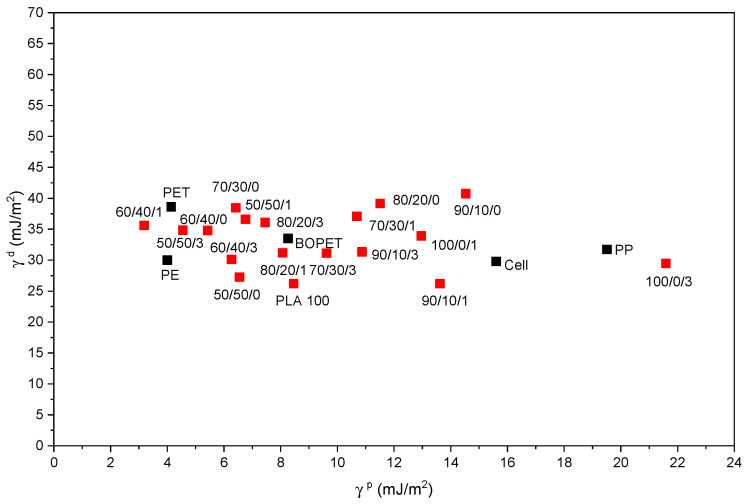
Dispersive and polar components of surface free energy for the produced PLA/PCL/SiO_2_ blends (red squares) and commercial materials (black squares): transparent polyethylene terephthalate (PET), biaxially oriented polyethylene terephthalate (BOPET), and polypropylene (PP). The results for the dispersive and polar components of surface free energy for polyethylene (PE) and cellophane (Cell) were adopted from [63] and [64], respectively.

Figure 9 shows the surface properties of the blends produced in this study alongside those of other commercially available materials. The dispersive (γ^d^) and polar (γ^p^) components of surface free energy are presented for neat PLA and PLA/PCL blends, both without nanoparticles and with 1 and 3 wt% SiO_2_. Five commercially available polymer materials, primarily used in the food packaging industry, are also included for comparison: transparent polyethylene terephthalate (PET), biaxially oriented polyethylene terephthalate (BOPET), polypropylene (PP), polyethylene (PE), and cellophane (Cell).

The results for the dispersive and polar components showed that the dispersive component of the surface free energy of selected commercial polymer materials ranges from 29.8 to 38.64 mJ/m^2^, while the polar component ranges from 4 to 19.5 mJ/m^2^. For the produced polymer blends, the lowest value of the dispersive component (26 mJ/m^2^) was measured for neat PLA, and the highest value (40.7 mJ/m^2^) was measured for the PLA/PCL 90/10 sample. The polar component ranges from 3.2 mJ/m^2^ (measured for the PLA/PCL/SiO_2_ 60/40/1 sample) to 21.5 mJ/m^2^ (measured for PLA with 3 wt% silica). One can conclude that the prepared polymer blends have dispersive and polar components of surface free energy within the range of commercial materials. The exception is PLA/PCL/SiO_2_ 60/40/1, which had a slightly lower polar component of surface free energy, and PLA with 3 wt% silica, which showed a slightly higher polar component of surface free energy compared to commercial materials.

#### 3.4.3. Adhesion Parameters Between PLA/PCL/SiO_2_ Blends and Flexographic Ink

**Figure 10:**
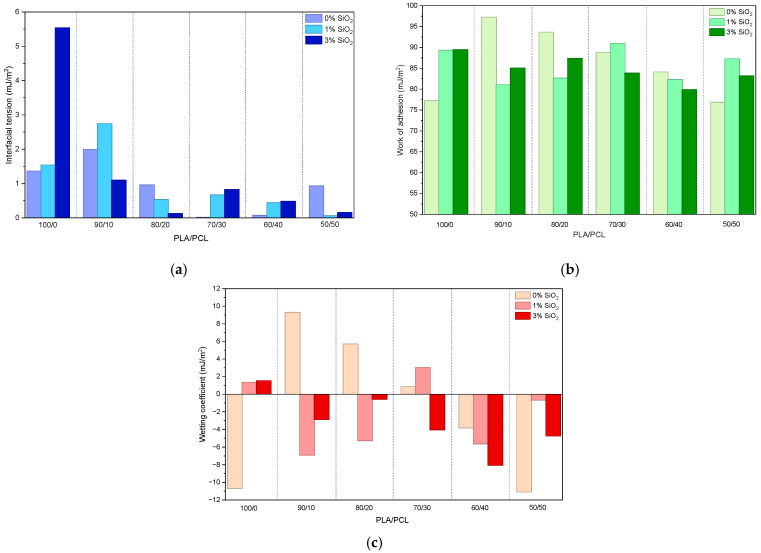
Adhesion parameters between PLA/PCL/SiO_2_ blends and flexographic ink: (**a**) interfacial tension; (**b**) work of adhesion; (**c**) wetting coefficient.

The interfacial interactions between PLA/PCL/SiO_2_ blends and the dried flexographic UV-curable printing ink layer were evaluated using interfacial tension (γ_12_), thermodynamic work of adhesion (W_12_), and wetting coefficient (S_12_), which are presented in Figure 10a–c, respectively (Table S10).

These parameters were calculated based on the SFE components of the polymer blends and the ink layer, which had a calculated total SFE of 43.99 mJ/m^2^, and was dominated by a dispersive SFE component (38.16 mJ/m^2^) with a relatively low polar SFE component (5.83 mJ/m^2^).

The interfacial tension values between the polymer blends and the ink were generally low (Figure 10a), ranging from 0.01 to 5.54 mJ/m^2^. Such low γ_12_ values indicate good compatibility between the ink and most examined polymer blend surfaces. Specifically, neat PLA (sample 100/0/0) exhibited a relatively low interfacial tension in interaction with ink (1.37 mJ/m^2^), which increased noticeably with the addition of 3 wt% SiO_2_ (sample 100/0/3, 5.54 mJ/m^2^). This increase suggests that excessive surface enrichment with polar silanol groups can reduce compatibility with the predominantly dispersive UV-curable flexographic ink. In contrast, the SiO_2_ concentration of 1 wt% resulted in only a marginal change in γ_12_. For PLA/PCL blends, particularly those with higher PCL contents, γ_12_ values approached zero (e.g., 70/30/0, 60/40/0, and 50/50/1), indicating near-ideal interfacial interaction. This behavior reflects the strong dispersive character of PCL, which aligns well with the dispersive-dominated SFE of the ink.

The calculated work of adhesion values (Figure 10b) ranged from 76.88 to 97.28 mJ/m^2^, indicating generally favorable adhesion across all polymer blend compositions. The highest W_12_ value was observed for the 90/10/0 blend (97.28 mJ/m^2^), suggesting an optimal interaction between the ink and a blend rich in PLA, containing a lower amount of PCL. In general, PLA/PCL blends (without SiO_2_) exhibited higher W_12_ values than their counterparts with 3 wt% SiO_2_ for most of the blends. This trend could be explained by the mismatch between the ink’s low polar SFE and the increased polarity introduced by SiO_2_. While silica enhanced surface wettability, it did not necessarily maximize adhesion to a largely dispersive ink system. At higher PCL contents, W_12_ tended to decrease slightly but remained relatively high (>80 mJ/m^2^), confirming that dispersive interactions dominate adhesion in these systems. The results again highlight that high wettability does not automatically translate into maximum adhesion strength when the surface free energy components are not well balanced.

The wetting coefficient provides direct insight into the spontaneous wetting of the ink on the polymer surface. Positive S_12_ values indicate thermodynamically favorable wetting, whereas negative values suggest partial wetting [65].

Several blend compositions exhibited positive S_12_ values, specifically PLA/PCL/SiO_2_ 100/0/1, 100/0/3, 90/10/0, 80/20/0, 70/30/1, and 70/30/0 (Figure 10c). These blends are characterized by either moderate PCL contents or low SiO_2_ concentration, i.e., conditions that favor a surface energy balance dominated by dispersive interactions. In contrast, most samples rich in SiO_2_ showed negative S_12_ values, indicating reduced spontaneous wetting. This occurrence underscores an important point: ink wetting and adhesion are influenced by SFE component matching rather than total SFE alone. The flexographic ink, with its low polar SFE component, spreads more effectively on surfaces with a similar dispersive-to-polar ratio.

When considered alongside water contact angle measurements (Figure 7), the adhesion data confirm that increasing surface hydrophilicity through SiO_2_ addition does not necessarily improve ink adhesion. While SiO_2_ significantly lowers water contact angles, particularly for the blends with a higher PLA/PCL ratio, it introduces polar surface functionalities that are poorly matched with the UV-curable flexographic ink’s surface chemistry.

Therefore, PLA/PCL/SiO_2_ blends with moderate PCL content and minimal or no SiO_2_ achieve a more favorable balance of SFE components in terms of the interactions with flexographic ink used in this research, leading to lower interfacial tension, higher work of adhesion, and positive wetting coefficients. These compositions, therefore, represent an optimal compromise between wettability control and printability. From a practical perspective, the results indicate that blends with higher PLA content, with 10–20 wt% PCL and without SiO_2_, offer more favorable conditions for UV-curable flexographic ink adhesion. Although SiO_2_ is effective in modifying surface wettability, its use should be carefully optimized when targeting printing applications that use inks dominated by dispersive forces.

Overall, the adhesion behavior of PLA/PCL/SiO_2_ systems is influenced by complex interactions between surface chemistry and morphology, filler-induced polarity, and polymer blend composition. These findings underscore the importance of considering all SFE components when designing biodegradable polymer packaging substrates for printing/coating applications.

### 3.5. Optical Density of PLA/PCL/SiO_2_ Blends and Printed Layer

**Figure 11:**
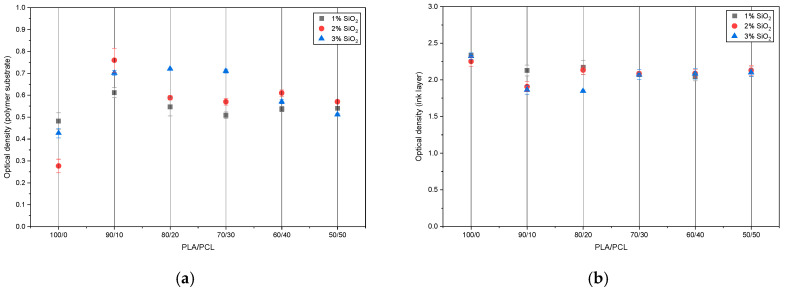
Optical density of (**a**) PLA/PCL/SiO_2_ substrates and (**b**) ink layer on PLA/PCL/SiO_2_ substrates.

Measuring the optical density of a polymer substrate is important because it indicates how much light the material absorbs or blocks, which affects print appearance and quality. Optical density measurements also support quality control by detecting variations in material composition, pigmentation, or surface treatment that can influence printing performance. Figure 11a (Table S11) shows the optical density results for the measured polymer substrates, conducted in reflection mode. PLA with 1 wt% SiO_2_ had the lowest density (0.28), while the highest density was measured in the PLA/PCL/SiO_2_ 90/10/1 sample (0.78). The addition of the PCL component increased the density, regardless of the PCL amount. Adding SiO_2_ nanoparticles to the blends caused a slight change in optical density. In most samples, the optical density slightly increased with the addition of SiO_2_ to the PLA/PCL blends.

The thickness of the ink applied to the polymer substrates was measured with a thickness gauge and found to be 5.13 µm on average. Figure 11b presents the absolute optical density values of the printed layers measured in reflection mode. The density values ranged from approximately 1.85 to 2.35, regardless of the polymer substrate type. The addition of nanoparticles caused a slight, insignificant decrease in print density, likely due to light scattering from silica particles in the ink layer, which reduced the effective absorption of the pigment (most visible on samples PLA/PCL/SiO_2_ 90/10/3 and 80/20/3). According to the Flexographic Technical Association, the absolute density value depends on several parameters. For film substrates, a value of 1.5, as a starting point for printing trials with black ink, ensures optimal density [55]. The obtained optical density values of black ink coated on the newly produced PLA/PCL and PLA/PCL/SiO_2_ substrates were higher than the recommended value and are comparable with other publications [28,56]. Based on these results, it can be assumed that PLA/PCL/SiO_2_ substrates are optimal for the application of UV-curable flexographic inks.

## 4. Conclusions

In this research, biodegradable PLA/PCL blends modified with silica nanoparticles were systematically investigated regarding surface morphology, roughness, wettability, surface free energy, adhesion with flexographic UV-curable ink, and optical properties.

The results showed that both blend composition and SiO_2_ content significantly influenced surface structure and functional performance relevant to printing applications. SEM and roughness analyses revealed that incorporating PCL into the PLA matrix increased surface irregularities due to the immiscibility and phase-separated morphology of PLA/PCL blends. The formation of PCL-rich domains led to higher R_a_, R_z_, and R_max_ values and more pronounced surface profiles, particularly at higher PCL contents. In contrast, the addition of silica nanoparticles significantly reduced surface roughness and suppressed phase separation. Cross-sectional SEM images confirmed that SiO_2_ acted as an effective physical compatibilizer, refining the blend morphology and promoting finer dispersion of the PCL phase, likely through interfacial localization and restriction of polymer chain mobility.

Surface wettability and surface free energy were strongly affected by silica addition. However, the influence of SiO_2_ diminished at higher PCL contents, indicating competition between silica-induced hydrophilicity and PCL surface segregation. The non-linear trends in surface free energy highlighted the combined influence of filler distribution, blend morphology, and surface composition.

Adhesion analysis showed that optimal ink–substrate interactions were not determined by total surface free energy or wettability alone, but rather by the balance between dispersive and polar components. While SiO_2_ generally lowered water contact angles on polymer blend surfaces, changes in dispersive-to-polar surface free energy ratio apparently reduced compatibility with the predominantly dispersive UV-curable flexographic ink, leading to increased interfacial tension and reduced, or negative, wetting coefficients. The most favorable adhesion parameters—low interfacial tension, high work of adhesion, and positive wetting coefficients—were achieved for PLA/PCL blends containing moderate PCL contents (10–20 wt%) with minimal or no SiO_2_.

Optical density measurements confirmed that all the investigated substrates enabled printing, with printed ink layers exhibiting optical densities well above industrial requirements for flexographic printing. Neither PCL incorporation nor silica addition adversely affected print density, demonstrating the suitability of these biodegradable substrates for printing applications.

Overall, this work demonstrated that PLA/PCL/SiO_2_ systems offer a versatile platform for tailoring surface properties through controlled adjustment of polymer blend composition and nanoparticle content. While SiO_2_ is effective for enhancing surface hydrophilicity and smoothing surface morphology, its concentration must be carefully optimized for printing applications dominated by primarily dispersive ink–substrate interactions. The findings provide valuable guidelines for the development of sustainable, printable polymer substrates for packaging and related applications.

## Figures and Tables

**Table 1:** Surface energy components of probe liquids.

Probe Liquid	γ_l_ ^d^ (mJ/m^2^)	γ_l_ ^p^ (mJ/m^2^)	γ_l_ ^total^ (mJ/m^2^)
Distilled water, γ = 2.0 μS/cm	21.80	51.00	72.80
Glycerol (purity 99.5%)	30.00	34.00	64.00
Diiodomethane (purity 99.0%)	50.80	0.00	50.80

## Data Availability

The original contributions presented in this study are included in the article/Supplementary Material. Further inquiries can be directed to the corresponding authors.

## Supplementary Materials

The following supporting information can be downloaded at: https://www.mdpi.com/2073-4360/18/3/422/s1, Table S7: Contact angle of water on polymer blends; Table S8: SFE of polymer blends; Table S10: Adhesion parameters; Table S11: Density of polymer substrates and ink layer.
